# Composition of Higher Alcohols in Different Alcoholic Beverages and Their Metabolic Dynamics in Bama Pigs

**DOI:** 10.3390/foods13203316

**Published:** 2024-10-18

**Authors:** Xiaonian Cao, Yunfei Hou, Qingqing Liu, Qian Yang, Min Liu, Haixu Lin, Qingxi Ren, Jian Mao

**Affiliations:** 1 Luzhou Laojiao Co., Ltd., Luzhou 646000, China; caozengjia@163.com (X.C.); liuqq1@lzlj.com (Q.L.); liumin2@lzlj.com (M.L.); 2 School of Food Science and Technology, Jiangnan University, Wuxi 214122, China; 7220112017@stu.jiangnan.edu.cn (Y.H.); 6220111132@stu.jiangnan.edu.cn (Q.Y.); darling_iu@163.com (H.L.); maojian@jiangnan.edu.cn (J.M.)

**Keywords:** alcoholic beverages, higher alcohol, Bama pig, pharmacokinetics, alcohol metabolism

## Abstract

The unique flavour contribution of higher alcohols in alcoholic beverages has received growing attention; however, there is a dearth of information on their in vivo metabolic kinetics. In this study, the composition and content of higher alcohols in different alcoholic beverages from Chinese *Baijiu* and *Lujiu* were studied via in vivo analysis using Bama pigs to elucidate the mechanisms for intoxication of alcohol in vitro and in drinkers. Direct injection combined with gas chromatography–mass spectrometry (GC-MS) were used to accurately quantify a total of 14 higher alcohols in five alcoholic beverages. Based on the external standard method, a total content of 289.37–938.33 mg/L was detected, mainly 1-butanol, 3-methyl-1-butanol, 1-hexanol, 2-methyl-1-propanol and 2-butanol. Then, headspace solid-phase microextraction (HS-SPME) and solid-phase extraction (SPE) combined with GC-MS analysis strategy, respectively, were adopted to continuously monitor the changes in the concentrations of ethanol and 11 higher alcohols in the blood within 24 h after gavage of different alcoholic beverages, and the key pharmacokinetic parameters were analysed. The peak concentration (C_max_) and area under curve (AUC) of blood higher alcohols were significantly lower than those of ethanol (*p <* 0.05), accompanied by a later peak time (T_max_) and a larger apparent clearance rate (CL_F), and there were certain differences between the same higher alcohols in different alcoholic beverages and between different higher alcohols in the same alcoholic beverage. This work provides valuable insights into the metabolism of alcoholic beverages.

## 1. Introduction

Higher alcohols, also known as “fusel alcohols” or “fusel oils”, is a general term for high-boiling monohydric alcohols containing more than two carbon atoms. These alcohols mainly include n-propanol, n-butanol, isobutanol, isopentanol, n-hexanol and phenylethyl alcohol, which are widely found in various types of alcoholic beverages such as *Baijiu*, *Huangjiu*, beer and wine [1,2]. Higher alcohols occur naturally in alcoholic beverages as by-products of alcoholic fermentation by *Saccharomyces cerevisiae*, and their biosynthesis mainly includes the Ehrlich pathway for amino acid catabolism and the Harris pathway for sugar metabolism. The main factors affecting their production include the content and type of carbon and nitrogen sources in the fermentation raw materials, the carbon–nitrogen ratio, fermentation conditions and brewing technology, etc. [3,4,5]. An appropriate amount of higher alcohols contributes to the mellow taste and delicate aroma of alcoholic beverages, but an imbalanced or excessive amount will generate undesirable flavours and easily cause hangover symptoms such as thirst, dizziness and headaches after consumption [6,7]. In addition, studies have shown that the anaesthetic effect of higher alcohols is stronger than that of ethanol at the same dose [8,9,10]. It mainly inhibits the human nerve centre and causes damage to the sympathetic nerves and visual nerves, and its oxidative decomposition rate is slower than that of ethanol in the human body, accompanied by a longer metabolic residence time in the body [9,10]. Therefore, different compositions, contents and proportions of higher alcohols not only bring various unique aromatic profiles to alcoholic beverages, but are also an important reason for the differences in the speed of metabolism, the level of intoxication and the degree of hangover after consuming different alcoholic beverages [7]. Overall, the unpleasant feeling caused by higher alcohols after drinking has attracted widespread attention from many researchers. However, studies on drunkenness and hangover mainly focus on ethanol metabolism, while the in vivo metabolic patterns of different higher alcohols in alcoholic beverages are still unclear.

There is currently no unified standard for limiting the content of higher alcohols in alcoholic beverages. Replete studies have shown that the content of higher alcohols is generally very limited in high-quality alcoholic beverages and abnormally high in low-quality alcoholic beverages. Specifically, the content of higher alcohols is <50 mg/L in high-quality beer, and its optimal content < 300 mg/L in wine, 600–2500 mg/L in Xiaoqu liquid-fermented Chinese *Baijiu*, 500–1800 mg/L in solid-fermented Chinese *Baijiu* and 80–540 mg/L in Chinese *Huangjiu* [7,10,11]. The qualitative and quantitative methods for higher alcohols in alcoholic beverages are mainly through pretreatment such as direct injection, liquid–liquid extraction (LLE) and headspace solid-phase microextraction (HS-SPME) combined with gas chromatography–mass spectrometry (GC-MS) [12,13]. However, the accurate detection of higher alcohols in plasma after drinking alcoholic beverages is still challenging due to their low content and complex matrix [7]. In addition, due to the ethical and legal issues in biomedical research involving human participants, a large part of alcohol metabolism-related investigations are conducted by non-human animals as experimental models [14], including mice [15], rats [16], zebrafish [17], *Caenorhabditis elegans* [18], etc. However, due to their small body size, low blood volume and difficulties in continuous blood collection, they are restricted in their application to the continuous tracking of alcohol metabolism blood concentration [19]. Bama pigs are an attractive alternative model for human alcoholism due to their behavioural, anatomical and physiological similarities to humans, and are often used to bridge the gap between rodents and humans in alcohol research [20].

*Baijiu* and *Lujiu* are two of the most traditional Chinese alcoholic beverages, playing an important role in people’s daily lives, and their different higher alcohol metabolism patterns are closely related to the perception after drinking. In the early stage, a previous study developed a solid-phase extraction method combined with gas chromatography–mass spectrometry (SPE-GC/MS) analysis strategy for the accurate detection of higher alcohols in the blood after consuming alcoholic beverages [7]. Based on this previous study, in the current study, a total of five different alcoholic beverages (four representative *Baijiu* and one *Lujiu*) were selected for the composition and content analysis of higher alcohols. A continuous blood sampling model for Bama pigs was established to monitor their blood’s higher alcohol concentrations at different time points (0, 15, 30, 60, 90, 120, 240, 480, 600 and 1440 min) after gavage of different samples. Subsequently, the blood higher alcohol concentration–time curve was established, and its key parameters of in vivo metabolic kinetics were analysed based on pharmacokinetic theory. This study is the first in a series of studies describing the continuous metabolism of higher alcohols in the blood, aiming to provide theoretical support and practical guidance for the development of high-quality alcoholic beverages.

## 2. Materials and Methods

### 2.1. Materials

Standards of higher alcohols (chromatographic grade), including 2-butanol, 2-methyl-1-propanol, 2-pentanol, 1-butanol, 3-methyl-1-butanol, 1-pentanol, 1-hexanol, 3-octanol, 1-heptanol, 2-nonanol, 1-octanol, 2-furan methanol, 1-decanol and 2-phenylethanol, were purchased from Sigma-Aldrich Co., Ltd. (Shanghai, China). Anhydrous ethanol, dichloromethane and other solvents of chromatographic grade were purchased from Aladdin Bio-Chem Technology Co., Ltd. (Shanghai, China). Methane (>99.9%) and helium (>99.999%) were purchased from XinXiYi Technology Co., Ltd. (Wuxi, Jiangsu, China). Bonded-phase polystyrene–divinylbenzene (PS-DVB, 250 mg) cartridges were purchased from Sigma-Aldrich Co., Ltd. (Shanghai, China). Nong-flavoured *Baijiu* A1 (sorghum, maize, wheat, 50.8% by volume, Luzhou, Sichuan, China) and Chinese *Lujiu* A2 (a specific style of alcoholic beverage based on Chinese Baijiu A1 which includes tea extract, sorghum, maize, wheat, 50.8% by volume, Luzhou, Sichuan, China) were obtained from Luzhou Laojiao Co., Ltd. (Luzhou, Sichuan, China), and Nong-flavoured *Baijiu* B (sorghum, wheat, waxy rice, maize, corn, 52.0% by volume, Deyang, Sichuan, China), Jiang-flavoured *Baijiu* C (sorghum, maize, 53.0% by volume, Luzhou, Sichuan, China) and D (sorghum, maize, 52.0% by volume, Zunyi, Guizhou, China) were purchased from a local market. *Lujiu* A2 was first obtained by adding tea extract for infusion fermentation (semi-fermentation) and then utilising a redistillation process. *Baijiu* A1, B, C and D were obtained from grains by processes such as brewing, fermentation, distillation and ageing. Other detailed information on the alcoholic beverages is shown in Table S1.

### 2.2. Animal Experiments

Approvals for animal experiments were obtained from the Institutional Animal Care and Use Committees (IACUC) of China (approval number: SYXK 〈Jin〉 2019-0006), and the animal experiments were conducted in strict accordance with the guidelines of the animal ethics committee at Howe Biotechnology Co., Ltd. (approval number: 2023010901, approval date: 9 January 2023, Tianjin, China). Thirty-six nine-month-old Bama pigs (*n* = 36, 30–35 kg, qualified number: SCXK 〈Jin〉 2020-0002) were purchased from Bainong Laboratory Animal Breeding Technology Co., Ltd. (Tianjin, China), and housed individually under controlled conditions (temperature 18–25 °C, 55–75% humidity, and 12 h light/dark cycle). After 7 days of acclimatisation, the Bama pigs were distributed to two experiments including gavage dose optimisation and higher alcohol metabolism studies. Different alcoholic beverages were gavaged once to the Bama pigs via a pressurised bulb in a gavage apparatus, which was a rubber tube with a funnel at one end where the other end went directly into the stomach of Bama pigs. In the gavage dose optimisation experiment, Bama pigs which had been fasted for 6 h were weighed and then gavaged with a dose gradient of 400–700 mL/pig (50% alcohol by volume, *n* = 6) according to body weight [21,22]. The optimal gavage dose was determined as the maximum acceptable dose under the premise of ensuring sufficient blood collection and normal physiological status. In the higher alcohol metabolism experiment, Bama pigs were randomly divided into five groups (*n* = 6) and were administered with different alcoholic beverages including *Baijiu* A1, *Lujiu* A2, *Baijiu* B, *Baijiu* C and *Baijiu* D at the optimal dose. Venous blood collection before gavage was recorded as 0 min and was continuously collected in EDTA anticoagulation tubes at 15, 30, 60, 90, 120, 240, 480, 600 and 1440 min after gavage. Subsequently, blood samples were centrifuged (1000× *g*, 15 min) to obtain plasma and stored at −80 °C for analysis.

### 2.3. Analysis of Blood Ethanol Concentration in Bama Pigs

Blood ethanol concentrations of Bama pigs at different times after intragastric administration of different alcoholic beverages were analysed using a headspace solid-phase microextraction gas chromatography–mass spectrometry (HS-SPME-GC-MS) strategy [7]. Briefly, the plasma sample (1 mL) was transferred into a 20 mL gastight headspace vial and equilibrated at 70 °C for 5 min with a magnetic microstirring device at 500 rpm. Then, HS-SPME was carried out using a divinylbenzene/carboxen/polydimethylsiloxane fibre (DVB/CAR/PDMS; length: 2 cm; df: 50/30 µm; Supelco, Sigma-Aldrich, St. Louis, MO, USA) and an automatic type SPME holder obtained from Supelco (Sigma-Aldrich, St. Louis, MO, USA). After 30 min of adsorption on the SPME fibre, GC-MS analysis was performed using a Thermo Trace1300 gas chromatograph coupled to a Thermo ISQ7000 mass detector equipped with a TG-WAXMS chromatographic column (0.25 µm, 30 m × 0.25 mm, Thermo Fisher Scientific, Waltham, MA, USA). The column carrier gas helium was at a constant flow rate of 1.0 mL/min, and the split ratio was 10:1. The injection temperature was 220 °C, and the oven temperature programme was maintained at 35 °C for 4 min, after which it was increased to 240 °C at 40 °C/min and held for a further 2 min. Mass spectra were operated in electron impact (EI) mode at 70 eV, with a scan range of *m*/*z* 29, 44, 43, 31, 45 and 46. The temperatures of the ion source and transmission line were 230 °C and 250 °C, respectively. Identification of ethanol was achieved by matching retention time (RT) and mass spectra fragments, and absolute quantification was carried out using the external standard method in a selected ion monitoring (SIM) model (Table S2).

### 2.4. Analysis of Blood Higher Alcohol Concentration in Bama Pigs

Blood higher alcohol concentrations of Bama pigs at different times after intragastric administration of different alcoholic beverages were analysed using SPE-GC/MS according to Liu, Cao et al. (2024) with some modifications [7]. The plasma sample was passed through a 0.2 µm aqueous membrane, 3 mL of the sample was loaded onto a PS-DVB cartridge (pre-activated with 6 mL dichloromethane, 6 mL methanol and 6 mL ultrapure water) and the sample was eluted with 3 mL ultrapure water and 7 mL of dichloromethane to yield higher alcohols. The eluate was dehydrated with anhydrous Na_2_SO_4_, evaporated under nitrogen and dissolved with 0.2 mL of dichloromethane for further GC-MS analysis. The pretreated blood sample was separated by a DB-Wax column (0.25 µm, 30 m × 0.25 mm) in the mode of a non-split model at an injection temperature of 250 °C and the carrier gas was helium (99.999%) at a constant flow rate of 1.0 mL/min. The chromatographic programme was set at 40℃ (held for 2 min), raised to 230 °C at 7 °C /min and held for 10 min. The MS data were acquired in the SIM mode, with the solvent delay set at 4.2 min. Ion source and transmission line temperatures were maintained at 280 °C and 240 °C, respectively, with an EI mode of 70 eV. Higher alcohols were identified by comparison to retention time (RI) values and by comparison with the MS fragmentation patterns of the reference compounds. Quantification was performed in SIM mode with target masses of *m*/*z*, the regression equation and the linear range for each higher alcohol as shown in Table S2. All target masses of each higher alcohol were used for calibration by an external standard method to quantify plasma samples.

### 2.5. Pharmacokinetic Analysis

**Figure 1:**
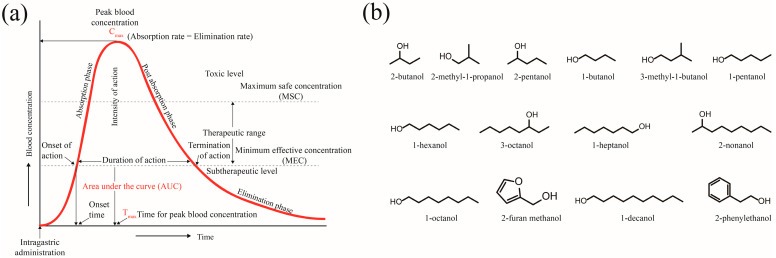
Classic blood drug concentration–time curve and chemical structure of higher alcohols. (**a**) Classic blood drug concentration–time curve. (**b**) Chemical structure of higher alcohols.

Pharmacokinetic parameters were calculated based on the one-compartment pharmacokinetic (PK) model according to the blood ethanol (or higher alcohol) concentration–time curve using Phoenix WinNonlin 8.4.0 (Pharsight, St. Louis, MO, USA). Model validation was carried out comprehensively through observation–prediction plots, residual–time plots and residual–prediction plots. Sensitivity analysis of model parameters was performed based on standard error (SD), coefficient of variation (CV%) and confidence interval width. The model was considered successful when the CV% between prediction and observation was less than 5% [23,24]. The following pharmacokinetic parameters were calculated (Figure 1): peak concentration (C_max_), time to peak (T_max_), absorption rate constant (K_01_), elimination rate constant (K_10_), area under the concentration–time curve (AUC) and clearance corrected for bioavailability (CL_F). Pharmacokinetic parameters were presented as heatmaps with normalisation.

### 2.6. Statistical Analysis

All experiments were carried out at least in triplicate and result data were expressed as means ± SD. Differences between groups were analysed by IBM SPSS Statistics (version 23.0, SPSS Inc., Chicago, IL, USA) using both analysis of variance (ANOVA) and Duncan post-tests using a significance level of *p* < 0.05.

## 3. Results and Discussion

### 3.1. Composition of Higher Alcohols in the Alcoholic Beverages

Higher alcohols are important volatile compounds in many alcoholic beverages that affect sensory attributes and post-drinking comfort [25]. A total of 14 higher alcohols, including 10 straight-chain fatty alcohols, 2 branched-chain fatty alcohols, 1 heterocyclic alcohol (2-furan methanol) and 1 aromatic alcohol (2-phenylethanol), were quantified from five alcoholic beverages by direct injection combined with GC-MS using the external standard method (Table 1).

The total amount of higher alcohols in the five alcoholic beverages ranged from 289.37 to 938.33 mg/L, which concurs with the existing literature [5,10]. Among them, 1-butanol, 3-methyl-1-butanol, 1-hexanol, 2-methyl-1-propanol and 2-butanol were the main components, accounting for more than 80% of the total higher alcohol content in each alcoholic beverage. Different higher alcohols provide different aroma characteristics. 1-butanol has a strong ethanol smell and a faint jasmine fragrance, 3-methyl-1-butanol has a typical higher alcohol aroma, 1-hexanol has a strongly fruity aroma, 2-methyl-1-propanol has a strong ethanol smell and a fatty aroma and 2-butanol has a weak fragrance [1,26,27]. These higher alcohols were one of the important components of the unique flavour skeleton of the studied alcoholic beverages, and their composition in each wine sample varied greatly among samples. Samples A1 and A2 both presented similar higher alcohol profile, with the total amount being only about one-third of the other three alcoholic beverages. Controlling the content of higher alcohols and the purity of the base liquor are the prerequisites for the production of high-quality *Lujiu*. Higher alcohols are mainly regulated by adding an appropriate amount of assimilable carbon and nitrogen sources, controlling suitable fermentation conditions, breeding high-quality yeast with low production of higher alcohols and post-treatment including activated carbon/macroporous resin adsorption and ultrasonic ageing [1,26]. The content of 1-butanol, 2-pentanol and 1-pentanol in the Nong-flavour *Baijiu* B was significantly higher than in other alcoholic beverages, while significantly a higher content of 3-methyl-1-butanol, 2-methyl-1-propanol, 2-butanol, 1-heptanol and 1-decanol was detected in the Jiang-flavoured *Baijiu* C and D (*p* < 0.05). Different concentrations and types of higher alcohols imparted an individual range of organoleptic attributes to each alcoholic beverage such as alcoholic, fruity, pungent, solvent-like and rose-like or floral aromas. In addition, the aroma importance of higher alcohols extends to other flavour aspects of alcoholic beverages by serving as ester precursors [28]. The contents of 3-octanol, 2-nonanol, 2-furfuryl alcohol and 2-phenylethanol were relatively low in all the studied alcoholic beverages. However, they were not mainly used to produce alcoholic aroma, but also contributed their unique flavours such as 3-octanol with a mushroom aroma, 2-nonanol with a fruity aroma and 2-phenylethanol with a honey-like aroma and rose-like aroma [5]. 2-Furfuryl alcohol, as a kind of heterocyclic alcohol, had a slight oily caramel aroma, and its content was relatively high in the Nong-flavoured *Baijiu* C; 2-phenylethanol, as a kind of aromatic alcohol, had a high boiling point and a low content in distilled liquors but a high content in fermented liquors such as Chinese *Huangjiu* and grape wine, usually exceeding 40 mg/L [7]. Overall, the composition and content of higher alcohols in various alcoholic beverages varied greatly due to different raw materials, processes and production areas.

### 3.2. Blood Ethanol and Higher Alcohol Compositions

**Figure 2:**
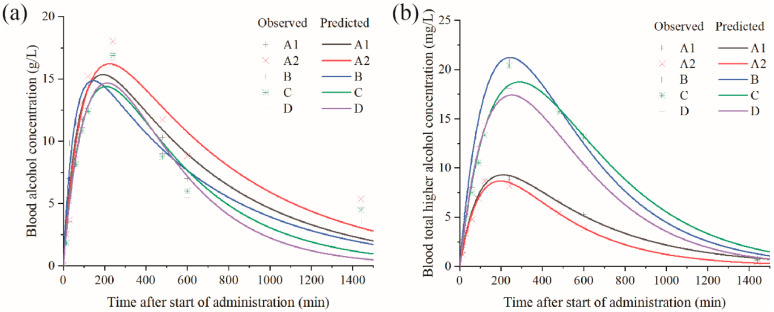
Blood ethanol and total higher alcohol concentration–time curves (C-T) of different alcoholic beverages. (**a**) Blood ethanol concentration–time curves (C-T) of different alcoholic beverages. (**b**) Total higher alcohol concentration–time curves (C-T) of different alcoholic beverages.

The blood concentration–time (C-T) curve can reflect the dynamic process of absorption, distribution, metabolism and excretion (ADME) of chemical components in the body [29]. Blood ethanol and higher alcohol concentrations of Bama pigs were detected within 24 h after intragastric administration of different alcoholic beverages using two pretreatment methods, HS-SPME and SPE, combined with GC-MS, respectively. Then, prediction and fitting were performed using a one-compartment PK model based on the average observed values of blood ethanol and total higher alcohols at different time points. The results are shown in Figure 2.

The C-T curves revealed several common characteristics (Figure 2a). The blood ethanol concentration increased to about 15 g/L immediately after intragastric administration of different alcoholic beverages, reflecting the absorption of ethanol from the gastrointestinal tract into the blood. As the concentration in the digestive tract decreased, the rate of absorption slowed down and eventually became equal to the clearance rate of ethanol metabolism and excretion, which indicated the starting point of the post-absorption phase. As long as the ethanol was no longer consumed, the blood ethanol concentration decreased at a relatively slow rate until it returned to the low level before intake, which was basically consistent with the features of the blood ethanol C-T curves reported in previous studies [29,30]. All the studied alcoholic beverages had similar absorption phases of blood ethanol but different elimination phases (Figure 2a). Ethanol, as a small uncharged polar molecule, was unable to easily cross the gut lumen by passive diffusion driven by the concentration and electric gradient of plasma without consuming energy, and its absorption rate depended mainly on the prevailing concentration gradient in accordance with Fick’s law [30]. The rate at which ethanol equilibrated between the water fraction of the blood and the extracellular fluids and tissue relied on the cross-sectional area of the local capillary bed and blood flow per gram of tissue. Organs with a rich blood supply, such as the brain and kidneys, equilibrate rapidly with ethanol in the blood, whereas bulky skeletal muscle with a relatively low blood flow to tissue mass ratio equilibrated more slowly [29,30]. Elimination of most ingested ethanol (90–98%) was carried out by oxidative metabolism primarily in the liver. A small portion (<1%) was removed from the body via conjugation to -OH groups to form the non-oxidative metabolites ethyl glucuronide and ethyl sulphate. The remainder (2–8%) was eliminated, unchanged by renal filtration and urinary excretion, as well as small amounts (1–2%) being excreted from the lungs and skin via respiration and sweat, respectively. The equilibrium rate of ethanol between blood water and extracellular fluid and tissue depends on the cross-sectional area of the local capillary bed and the blood flow per gram of tissue. Organs rich in fluid supply such as the brain and kidneys quickly reached equilibrium with ethanol in the blood, while large skeletal muscles with a low blood flow to tissue mass ratio had a slower equilibrium rate. Most of the ingested ethanol (90–98%) was excreted from the body through oxidative metabolism mainly in the liver. A small part (<1%) combined with -OH groups formed the non-oxidative metabolites ethyl glucuronide and ethyl sulphate. The remaining dose (2–8%) was eliminated through renal filtration and urinary excretion. A small amount of ethanol (1–2%) was also excreted from the lungs and skin through breathing and sweat. Excretion was a first-order process; thus, proportionally more ethanol was eliminated when larger doses or higher concentrations were reached in the blood [29,30,31]. Alcohol dehydrogenase (ADH) and acetaldehyde dehydrogenase (ALDH) were the main enzyme systems for alcohol metabolism, and both were polymorphic enzymes that existed in multiple molecular forms. The catalytic activity and substrate specificity of various isozymes were different, and higher alcohols had greater affinity for enzymes than ethanol [10,30]. Therefore, the composition and content of higher alcohols in different alcoholic beverages may be the main reason for the difference in blood ethanol clearance.

The C-T curves of blood total higher alcohols were similar to that of ethanol, but varied greatly among the different alcoholic beverages. The C-T curve of *Lujiu* A2 was slightly lower than that of *Baijiu* A1, and both were obviously lower than those of *Baijiu* B, C and D (Figure 2b). Studies have suggested that the dose and speed of administration are important considerations when discussing the variability of the peak value of the C-T curve, clearance time and the area under the curve [31,32]. In the present study, the speed of drinking was the same, and the difference in the C-T curves of blood total higher alcohols after intragastric administration of the alcoholic beverages were mainly related to their initial higher alcohol content. It is worth noting that *Lujiu* A2 corresponded to a lower C-T curve of total higher alcohols than that of *Baijiu* A1, though its initial total higher alcohol content was higher than the latter. This may be related to the addition of tea extracts during the processing of *Lujiu* A2, which increased its first-pass metabolism in the stomach and reduced the bioavailability of the ingested higher alcohols [33].

### 3.3. Pharmacokinetic Parameters of Ethanol and Total Higher Alcohols

The basic principles of ethanol pharmacokinetics were established in the 1930s, including the concept of zero-order elimination kinetics of blood ethanol and the distribution of the absorbed dose in total body water, and this series of issues has been widely studied [34]. Similar information is currently lacking for higher alcohols, and there is no systematic scientific understanding of their in vivo metabolic kinetics and first-pass metabolism [10]. Thus, in the present study, the mean PK parameters of blood ethanol and total higher alcohols based on a one-compartment model are summarised in Table 2.

PK parameter analysis showed that there were significant differences in the CL_F, K_01_, K_10_, C_max_, T_max_ and AUC of blood ethanol in different alcoholic beverages (*p* < 0.05, Table 2). CL_F was the apparent clearance, reflecting the body’s ability to clear ethanol. AUC was the area under the C-T curve, which was an important indicator for evaluating the degree of ethanol absorption, and the reciprocal of the initial dose [35]. A significantly lower AUC of blood ethanol, accompanied by a higher CL_F, was found in beverage group A1 and A2 compared to groups B, C and D. This indicated that beverage groups A1 and A2 had lower ethanol absorption and a higher clearance rate than the other beverages. Moreover, the ethanol in beverage groups A1 and A2 was less absorbed and eliminated faster, which was also reflected in the low absorption rate constant K_01_ and larger elimination rate constant K_10_ in beverages A1 and A2 compared with beverage groups B, C and D. These results indicate that the relative bioavailability of ethanol in beverage groups A1 and A2 may be lower despite the same total amount of ethanol administered in all the studied beverages. Mitchell, Teigen and Ramchandani (2014) also found that blood alcohol metabolism varied greatly depending on the type of alcoholic beverage, as well as the rate and total amount of alcohol intake [35]. They considered this to be related to the different apparent distribution volumes and bioavailability of different alcoholic beverages in the body. In addition, the C_max_ of blood ethanol in Bama pigs was similar, which was about 15 g/L after intragastric administration of all the beverages. However, the peak time T_max_ differed greatly between the groups, with *Baijiu* B being the shortest and *Lujiu* A2 being the longest peak time of 223.28 min. The delay in the T_max_ of *Lujiu* A2 may be related to the lower content of higher alcohols in the base liquor and the tea extracts added during post-processing. Previous studies found that tea extracts promoted alcohol metabolism by decreasing serum alanine aminotransferase and aspartate aminotransferase activities and inhibiting cytochrome P450 2E1 expression [36]. They were also reported to protect against chronic alcohol-induced fatty liver disease by ameliorating oxidative stress and inflammation [37].

The C_max_ and AUC of the total higher alcohols in the blood after administration of the same alcoholic beverage were significantly lower than those of ethanol (14–17 g/L vs. 8–22 × 10^−3^ g/L), and there were also significant differences among different alcoholic beverages (*p* < 0.05). This was because the total higher alcohol content in each beverage was significantly lower than that of ethanol, with the former only 1/2000–1/400 of the latter, and the initial higher alcohol content varied greatly (Table 1). In addition, the peak time T_max_ of blood total higher alcohols was shorter than that of ethanol and was accompanied by a larger CL_F. On the one hand, higher alcohols such as 2-butanol and 2-methyl-1-propanol exhibited poor hydrophilicity, which limited their distribution rate in the body [38]. On the other hand, despite the greater affinity of higher alcohols to the enzyme, the metabolism of higher alcohols was suppressed by excess ethanol supply; a similar phenomenon has been observed in previous studies [10,38]. It is also worth noting that the AUC of total higher alcohols in the blood was significantly lower after intragastric administration of *Lujiu* A2 compared to the base *Baijiu* A1 despite the initial total higher alcohol content of the former being slightly higher than that of the latter; that is, the total bioavailability of higher alcohols in beverage group A2 was relatively lower. Park, Kim, Hwang, Bae, Bae and Song (2013) found that gallated catechins, as important components of green tea extract, played a key role in prolonging the absorption inhibition time and reducing alcohol intake [39]. Therefore, the development of *Lujiu* with health-preserving functions by integrating medicinal and edible active substances into traditional *Baijiu* is receiving a lot of attention.

### 3.4. Pharmacokinetic Parameters of Individual Higher Alcohols 

Richardson’s law pointed out that the toxicity and potency of aliphatic alcohols increased with the length of carbon chains, and that the oxidation rates of higher alcohols were different from those of ethanol, but this is controversial [10]. The concentrations of individual higher alcohols in the blood were measured using SPE-GC/MS at different times after intragastric administration of different alcoholic beverages to Bama pigs. The key parameters of the individual higher alcohol metabolism kinetics were analysed based on a one-compartment PK model, as shown in Table 3.

A total of eleven higher alcohols were detected in the blood of Bama pigs after gavage with five alcoholic beverages, while 3-octanol, 1-heptanol and 2-furan methanol were not detected due to their low levels and 2-pentanol was only detected in beverage group B. 2-Pentanol had an asymmetric chiral carbon atom and existed in two different stereoisomers (R) and (S), which may cause nausea, headache, vomiting and anaesthesia when ingested in excess [9,40]. Table 1 showed that the content of 2-pentanol in *Baijiu* B was significantly higher than that in the other beverages (121.63 vs. 3.79 mg/L, *p* < 0.05), which may be one of the reasons for the severe alcohol intoxication exhibited by Bama pigs administered with *Baijiu* B. In terms of the PK parameters of individual higher alcohols, there were significant differences between the different beverages or between different higher alcohols in the same beverage (Table 3, Figure S1). In terms of the PK parameters of individual higher alcohols, there were significant differences between the beverages or between different higher alcohols in the same beverage (*p* < 0.05, Table 3). Specifically, the initial oral dose did not differ significantly (*p* > 0.05) between the groups, but the T_max_ of blood 2-nonanol in beverage group A1 was higher than that in group D. The T_max_ of blood 2-phenylethanol in beverage group A1 was also higher than that in groups B and C. This may be attributed to other components in different beverages such as peptides, aldehydes and biogenic amines. Some peptides affect alcohol metabolism by activating liver ADH and ALDH [41]. Moreover, aldehydes and biogenic amines have a synergistic effect when combined with ethanol, thereby affecting metabolism in the body and the rate of intoxication [42]. This may be the main reason for the different metabolism of blood higher alcohols after intragastric administration of the beverages. In addition to the differences between the beverage groups, the metabolism of individual higher alcohols in the same beverage also varied greatly. This may depend on the initial concentration of individual higher alcohols in the same group, or may be related to the molecular weight (carbon-chain length) and configuration [7,38]. For example, the initial concentrations of 2-nonanol and 1-octanol in *Baijiu* C were similar but the T_max_ of blood 2-nonanol was higher than that of 1-octanol, and the AUC and C_max_ were only about one-fourth of the latter. The protein structure and electrophilicity of alcohol dehydrogenases involved in the metabolism of alcohols were different, resulting in different substrate specificity for different higher alcohols. Higher alcohols with branches on the side chains may affect the activity of the enzyme and reduce its efficiency, which requires further experimental verification [7,10,43]. According to previous studies, impurities such as higher alcohols, methanol and aldehydes may be major causes of alcohol poisoning and hangovers, and drinking alcoholic beverages with high impurity content (such as Chinese *Huangjiu*, whiskey, tequila, etc.) was accompanied by more severe hangover symptoms than drinking beverages with low impurity contents [44]. However, the assessment of discomfort was mainly based on subjective descriptions such as headache and thirst, and there was a lack of evidence based on rigorous controlled trials. This work preliminarily explored the continuous metabolic pattern of blood higher alcohols in the body, and the results revealed that there were significant differences between the beverage groups, and there were also significant differences between individual higher alcohols in the same beverage. These findings may be helpful for further evaluating the role of higher alcohols in alcohol metabolism and hangovers.

## 4. Conclusions

This study investigated the higher alcohol composition and content in different alcoholic beverages and their in vivo metabolism. A total of 14 higher alcohols were accurately quantified in the beverages, with 1-butanol, 3-methyl-1-butanol, 1-hexanol, 2-methyl-1-propanol and 2-butanol being the main forms, accounting for more than 80% of the higher alcohol content. After intragastric administration, 11 higher alcohols were accurately quantified in the blood of Bama pigs, and the metabolic pattern of blood higher alcohols shared some common characteristics with that of ethanol, such as a nearly linear absorption–distribution period and a slow metabolism–excretion period. Pharmacokinetic analysis clarified that there were certain differences in the in vivo metabolic patterns of the same higher alcohol between different alcoholic beverages and between different higher alcohols in the same alcoholic beverage, and the supplementation of some natural extracts (such as tea) played a positive regulatory role in accelerating the metabolism of alcohol and higher alcohols. These findings not only provide theoretical support for a new perspective on evaluating the quality of alcoholic beverages, but also offer practical guidance for better understanding the specific effects of higher alcohols on intoxication and hangover after binge drinking, which require more extensive research in combination with multiple factors such as dietary patterns.

## Figures and Tables

**Table 1:** Composition of higher alcohols in different alcoholic beverages.

No.	Retention Time (min)	Compounds	CAS	Quantitation (mg/L)				
				A1	A2	B	C	D
1	3.82	2-butanol	78-92-2	15.43 ± 0.57 ^d^	17.62 ± 0.44 ^d^	49.87 ± 1.84 ^c^	82.16 ± 3.48 ^b^	115.04 ± 8.39 ^a^
2	4.59	2-methyl-1-propanol	78-83-1	24.25 ± 2.04 ^d^	21.51 ± 1.54 ^d^	78.27 ± 0.59 ^c^	202.55 ± 2.19 ^a^	146.28 ± 2.27 ^b^
3	4.97	2-pentanol	6032-29-7	1.65 ± 0.04 ^b^	0.57 ± 0.03 ^b^	121.63 ± 2.57 ^a^	3.79 ± 0.71 ^b^	0.86 ± 0.04 ^b^
4	5.45	1-butanol	71-36-3	110.97 ± 0.68 ^b^	108.91 ± 0.37 ^b^	243.70 ± 2.68 ^a^	93.37 ± 3.88 ^c^	64.85 ± 1.78 ^d^
5	6.44	3-methyl-1-butanol	123-51-3	85.56 ± 0.57 ^d^	95.71 ± 0.68 ^d^	169.28 ± 5.90 ^c^	462.96 ± 22.49 ^a^	328.59 ± 9.03 ^b^
6	7.15	1-pentanol	71-41-0	5.17 ± 0.12 ^c^	5.13 ± 0.11 ^c^	25.75 ± 0.36 ^a^	9.88 ± 0.25 ^b^	2.57 ± 0.06 ^d^
7	8.93	1-hexanol	111-27-3	42.21 ± 0.67 ^de^	44.85 ± 0.32 ^cd^	149.72 ± 4.48 ^a^	55.19 ± 2.75 ^b^	33.93 ± 0.53 ^e^
8	9.58	3-octanol	20296-29-1	0.01 ± 0.00 ^b^	0.02 ± 0.00 ^b^	0.02 ± 0.00 ^b^	0.10 ± 0.01 ^a^	0.10 ± 0.00 ^a^
9	10.84	1-heptanol	111-70-6	1.46 ± 0.04 ^c^	2.09 ± 0.07 ^c^	2.46 ± 0.04 ^c^	17.83 ± 0.21 ^b^	23.27 ± 1.31 ^a^
10	12.07	2-nonanol	628-99-9	0.12 ± 0.01 ^c^	0.85 ± 0.01 ^b^	0.14 ± 0.00 ^c^	0.99 ± 0.06 ^a^	0.12 ± 0.01 ^c^
11	12.83	1-octanol	111-87-5	0.43 ± 0.01 ^c^	0.38 ± 0.02 ^c^	1.49 ± 0.01 ^a^	1.12 ± 0.02 ^b^	0.40 ± 0.01 ^c^
12	14.97	2-furan methanol	98-00-0	0.50 ± 0.00 ^c^	0.44 ± 0.01 ^c^	0.70 ± 0.02 ^b^	1.17 ± 0.04 ^a^	0.40 ± 0.01 ^c^
13	16.96	1-decanol	112-30-1	1.52 ± 0.07 ^d^	2.63 ± 0.11 ^d^	4.68 ± 0.05 ^c^	7.12 ± 0.17 ^b^	14.69 ± 0.88 ^a^
14	19.64	2-phenylethanol	60-12-8	0.07 ± 0.00 ^b^	0.08 ± 0.01 ^b^	0.06 ± 0.01 ^b^	0.10 ± 0.02 ^ab^	0.16 ± 0.01 ^a^
Total amount of higher alcohols				289.37 ± 2.09 ^d^	300.79 ± 2.12 ^d^	847.77 ± 13.02 ^b^	938.33 ± 28.71 ^a^	731.27 ± 3.93 ^c^

Note: Different letters in the same row indicate statistically significant differences between groups (*p <* 0.05).

**Table 2:** Pharmacokinetic parameters of blood ethanol and total higher alcohols after intragastric administration of different alcoholic beverages.

Categories	No.	CL_F (×10^−2^ L/min)	K_01_ (×10^−2^ L/min)	K_10_ (×10^−2^ L/min)	C_max_^#^	T_max_ (min)	AUC ^##^
Ethanol	A1	2.06 ± 0.43 ^Ba^	1.19 ± 0.34 ^Bb^	0.23 ± 0.05 ^ABa^	15.34 ± 1.03 ^ABb^	191.81 ±22.41 ^Ba^	9963.17 ± 2092.08 ^Ab^
	A2	2.26 ± 0.56 ^Ba^	1.00 ± 0.30 ^Ba^	0.32 ± 0.05 ^Ba^	16.20 ± 1.14 ^Bb^	223.28 ± 26.03 ^Cb^	9094.20 ± 2255.46 ^Ab^
	B	1.81 ± 0.33 ^Aa^	1.81 ± 0.55 ^Cb^	0.17 ± 0.05 ^Aa^	14.85 ± 1.08 ^ABb^	144.92 ± 22.04 ^Aa^	11,352.01 ± 2045.98 ^Ba^
	C	1.60 ± 0.26 ^Aa^	0.86 ± 0.45 ^ABa^	0.17 ± 0.05 ^Aa^	14.38 ± 1.30 ^Aa^	207.22 ± 28.93 ^BCa^	12,628.70 ± 2083.90 ^BCa^
	D	1.34 ± 0.25 ^Aa^	0.67 ± 0.13 ^Ab^	0.15 ± 0.05 ^Aa^	14.66 ± 1.63 ^Aa^	213.09 ± 34.11 ^BCa^	15,053.50 ± 2769.27 ^Cb^
Total higher alcohols	A1	2.16 ± 0.13 ^Aa^	0.88 ± 0.12 ^Ba^	0.22 ± 0.03 ^Aa^	9.30 ± 0.24 ^Aa^	209.55 ± 8.33 ^Aa^	6695.81 ± 404.88 ^Aa^
	A2	2.90 ± 0.25 ^Bb^	0.79 ± 0.24 ^ABa^	0.30 ± 0.01 ^ABa^	8.68 ± 0.35 ^Aa^	196.88 ± 12.27 ^Aa^	5186.64 ± 449.67 ^Aa^
	B	2.88 ± 0.19 ^Bb^	0.55 ± 0.17 ^Aa^	0.30 ± 0.01 ^ABb^	21.22 ± 0.57 ^Ca^	243.56 ± 8.88 ^Bb^	14,706.66 ± 977.91 ^Bb^
	C	3.19 ± 0.31 ^BCb^	1.03 ± 0.34 ^BCa^	0.35 ± 0.02 ^Bb^	18.74 ± 0.76 ^BCb^	288.96 ± 20.51 ^Cb^	14,717.24 ± 1409.17 ^Bb^
	D	3.07 ± 0.22 ^BCb^	0.96 ± 0.41 ^BCa^	0.38 ± 0.09 ^Bb^	17.40 ± 0.48 ^Ba^	251.79 ± 8.99 ^BCb^	11,917.79 ± 836.48 ^Ba^

Note: Different capital letters in the same column indicate significant differences (*p* < 0.05) between different alcoholic beverages in the same category (blood ethanol or total higher alcohols); different lowercase letters in the same column indicate significant differences (*p* < 0.05) between different categories (blood ethanol and total higher alcohols) in the same alcoholic beverage. # The C_max_ unit of ethanol was g/L, and that of total higher alcohols was mg/L; ## The AUC unit of ethanol was min·g/L, and that of total higher alcohols was min·mg/L.

**Table 3:** Pharmacokinetic parameters of individual higher alcohols after intragastric administration of different alcoholic beverages.

Categories	No.	CL_F (×10^−2^ L/min)	K_01_ (×10^−2^ L/min)	K_10_ (×10^−2^ L/min)	C_max_ (mg/L)	T_max_ (min)	AUC (min·mg/L)
2-butanol	A1	1.15 ± 0.24 ^Ac^	0.91 ± 0.12 ^Bb^	0.38 ± 0.08 ^Bb^	1.05 ± 0.10 ^ABb^	193.70 ± 29.62 ^Aab^	671.33 ± 142.53 ^Ab^
	A2	1.78 ± 0.38 ^Ab^	0.74 ± 0.23 ^Bab^	0.35 ± 0.34 ^Bab^	0.86 ± 0.09 ^Aab^	193.22 ± 31.24 ^Aab^	483.90 ± 44.59 ^Ab^
	B	1.81 ± 0.15 ^Acd^	0.85 ± 0.15 ^Bab^	0.20 ± 0.04 ^Aa^	1.76 ± 0.06 ^Bb^	222.34 ± 11.51 ^Ba^	1376.89 ± 114.13 ^Bc^
	C	2.53 ± 0.23 ^Bbc^	0.34 ± 0.03 ^Aa^	0.33 ± 0.09 ^ABab^	2.00 ± 0.06 ^Cc^	298.25 ± 13.87 ^Cc^	1621.88 ± 150.09 ^BCb^
	D	2.78 ± 0.25 ^Bb^	0.37 ± 0.13 ^Aa^	0.26 ± 0.15 ^Aa^	2.85 ± 0.32 ^Cb^	266.69 ± 10.33 ^BCbc^	2069.35 ± 187.48 ^Cc^
2-methyl-1-propanol	A1	1.63 ± 0.40 ^Acd^	0.84 ± 0.15 ^Cb^	0.38 ± 0.03 ^Ab^	1.22 ± 0.14 ^Ab^	194.70 ± 34.92 ^ABab^	745.83 ± 184.93 ^Bb^
	A2	3.17 ± 0.65 ^Bc^	0.23 ± 0.25 ^Aa^	0.44 ± 0.17 ^Bb^	0.84 ± 0.09 ^Aab^	130.92 ± 25.87 ^Aa^	339.50 ± 69.63 ^Ab^
	B	1.36 ± 0.14 ^Ac^	0.41 ± 1.93 ^Ba^	0.28 ± 0.02 ^Aab^	4.19 ± 0.17 ^Bc^	252.35 ± 13.33 ^Bab^	2873.04 ± 302.53 ^Cd^
	C	1.91 ± 0.31 ^Ab^	0.32 ± 0.03 ^ABa^	0.32 ± 0.05 ^Aab^	6.20 ± 0.69 ^Bd^	314.20 ± 13.38 ^Cc^	5294.71 ± 845.79 ^Ec^
	D	1.63 ± 0.17 ^Ab^	0.36 ± 0.09 ^ABa^	0.35 ± 0.01 ^Aab^	5.88 ± 0.22 ^Bc^	280.82 ± 13.89 ^Cbc^	4488.23 ± 470.17 ^Dd^
2-pentanol	B	54.37 ± 9.15 ^f^	0.37 ± 0.04 ^a^	0.35 ± 0.07 ^b^	0.15 ± 0.01 ^a^	278.15 ± 23.21 ^b^	111.86 ± 18.80 ^a^
1-butanol	A1	11.50 ± 1.58 ^Ac^	1.80 ± 0.39 ^Bc^	0.14 ± 0.03 ^Aa^	0.56 ± 0.03 ^BCb^	152.51 ± 16.91 ^Aa^	482.55 ± 66.27 ^Bb^
	A2	12.99 ± 1.97 ^Ad^	1.38 ± 0.37 ^Ab^	0.17 ± 0.05 ^Aa^	0.54 ± 0.03 ^BCa^	171.86 ± 20.03 ^ABa^	419.37 ± 63.62 ^Bb^
	B	12.49 ± 7.01 ^Ae^	0.98 ± 0.29 ^Ab^	0.23 ± 0.14 ^Aa^	0.66 ± 0.08 ^Ca^	193.37 ± 35.84 ^Ba^	451.53 ± 117.17 ^Bb^
	C	16.60 ± 2.40 ^Bd^	1.06 ± 0.45 ^Ac^	0.20 ± 0.12 ^Aa^	0.52 ± 0.04 ^Bab^	165.76 ± 20.51 ^ABa^	281.20 ± 40.61 ^Aab^
	D	15.38 ± 2.15 ^Bd^	1.85 ± 0.47 ^Bc^	0.20 ± 0.05 ^Aa^	0.32 ± 0.02 ^Aa^	135.47 ± 16.81 ^Aa^	210.85 ± 29.41 ^Aab^
3-methyl-1-butanol	A1	1.34 ± 0.15 ^Acd^	0.62 ± 0.20 ^Ba^	0.23 ± 0.08 ^Aab^	4.12 ± 0.18 ^Ad^	254.32 ± 15.96 ^ABb^	3200.25 ± 363.27 ^ABd^
	A2	2.06 ± 0.18 ^ABbc^	0.66 ± 0.19 ^Bab^	0.34 ± 0.15 ^ABab^	3.89 ± 0.16 ^Ab^	207.67 ± 12.42 ^Aab^	2319.30 ± 206.33 ^Ad^
	B	2.24 ± 0.17 ^Bd^	0.35 ± 0.09 ^Aa^	0.36 ± 35.62 ^ABb^	4.94 ± 0.23 ^Ac^	281.50 ± 34.76 ^Bb^	3778.03 ± 280.90 ^Bde^
	C	4.220.38 ^Cc^	0.34 ± 0.03 ^Aa^	0.37 ± 0.01 ^Bb^	7.18 ± 0.25 ^Bd^	280.77 ± 13.48 ^Bbc^	5483.73 ± 492.83 ^Cc^
	D	4.00 ± 0.52 ^Cc^	0.43 ± 0.09 ^Aa^	0.41 ± 0.05 ^Bb^	6.32 ± 0.33 ^Bc^	239.11 ± 15.74 ^Ab^	4107.86 ± 537.81 ^Cd^
1-pentanol	A1	1.91 ± 0.79 ^Be^	0.42 ± 0.05 ^Aa^	0.41 ± 0.05 ^ABb^	0.21 ± 0.03 ^Aa^	240.67 ± 42.14 ^Bb^	135.29 ± 56.06 ^Ba^
	A2	1.55 ± 0.37 ^ABb^	0.43 ± 0.04 ^Aa^	0.44 ± 0.04 ^Bb^	0.27 ± 0.03 ^Aa^	229.09 ± 48.45 ^ABab^	165.48 ± 39.10 ^Bab^
	B	1.05 ± 0.11 ^Ab^	0.98 ± 0.22 ^Cb^	0.22 ± 0.05 ^Aa^	1.77 ± 0.08 ^Cb^	194.98 ± 13.50 ^Aa^	1224.46 ± 119.89 ^Dc^
	C	1.29 ± 0.17 ^Aab^	0.71 ± 0.27 ^Bb^	0.25 ± 0.01 ^Aa^	0.53 ± 0.03 ^Bab^	228.57 ± 18.28 ^ABb^	381.69 ± 51.13 ^Cab^
	D	1.84 ± 0.32 ^Bb^	0.50 ± 0.07 ^ABa^	0.53 ± 0.07 ^Bb^	0.13 ± 0.01 ^Aa^	193.79 ± 28.03 ^Aab^	69.78 ± 12.18 ^Aa^
1-hexanol	A1	1.83 ± 0.20 ^ABe^	0.72 ± 0.47 ^Ab^	0.39 ± 0.05 ^Bb^	2.19 ± 0.13 ^ABc^	185.06 ± 16.55 ^ABab^	1150.92 ± 128.14 ^Ac^
	A2	1.79 ± 0.28 ^ABb^	0.49 ± 4.11 ^Aa^	0.46 ± 3.88 ^BCb^	2.20 ± 0.16 ^ABb^	209.78 ± 21.06 ^Bab^	1252.97 ± 198.04 ^Ac^
	B	1.54 ± 0.21 ^Ac^	0.63 ± 0.33 ^Aab^	0.29 ± 0.16 ^Aab^	7.27 ± 0.42 ^Cd^	228.19 ± 18.70 ^Ca^	4872.93 ± 676.53 ^Be^
	C	1.94 ± 0.53 ^Bb^	0.45 ± 0.08 ^Aa^	0.36 ± 9.92 ^Bb^	1.86 ± 0.20 ^Ab^	280.48 ± 43.77 ^Dbc^	1420.01 ± 385.54 ^Ab^
	D	1.56 ± 0.20 ^Ab^	1.30 ± 0.33 ^Bc^	0.20 ± 0.05 ^Aa^	1.55 ± 0.09 ^Ab^	169.90 ± 17.13 ^Aa^	1086.40 ± 139.26 ^Ac^
2-nonanol	A1	0.85 ± 0.13 ^Ab^	0.42 ± 0.02 ^Aa^	0.42 ± 0.02 ^BCb^	0.01 ± 0.00 ^Aa^	238.72 ± 15.98 ^ABb^	7.26 ± 1.14 ^Aa^
	A2	2.99 ± 0.43 ^Bc^	0.39 ± 0.33 ^Aa^	0.39 ± 0.04 ^Bb^	0.02 ± 0.00 ^Ba^	258.66 ± 36.83 ^Bb^	14.30 ± 2.04 ^ABa^
	B	0.51 ± 0.06 ^Aa^	0.35 ± 0.07 ^Aa^	0.36 ± 0.07 ^Bb^	0.02 ± 0.00 ^Ba^	282.56 ± 18.26 ^BCb^	13.43 ± 1.52 ^ABa^
	C	0.32 ± 0.02 ^Aa^	0.34 ± 173.16 ^Aa^	0.34 ± 0.01 ^Bab^	0.02 ± 0.00 ^Ba^	291.92 ± 139.52 ^Cbc^	15.41 ± 2.00 ^Ba^
	D	0.61 ± 0.15 ^Aa^	0.93 ± 0.15 ^Bb^	0.19 ± 0.03 ^Aa^	0.01 ± 0.00 ^Aa^	215.24 ± 11.48 ^Aab^	9.99 ± 0.83 ^Aa^
1-octanol	A1	0.67 ± 0.09 ^Aab^	1.24 ± 0.49 ^Cb^	0.16 ± 0.03 ^Aa^	0.04 ± 0.00 ^Aa^	125.81 ± 14.85 ^Aa^	32.11 ± 4.24 ^Aa^
	A2	0.79 ± 0.09 ^ABa^	1.28 ± 0.3 ^Cb^	0.20 ± 0.05 ^Aa^	0.03 ± 0.00 ^Aa^	171.75 ± 16.00 ^Ba^	24.07 ± 2.87 ^Aa^
	B	0.77 ± 0.09 ^ABab^	0.40 ± 0.2 ^Aa^	0.39 ± 0.02 ^Bb^	0.14 ± 0.01 ^Ba^	252.71 ± 13.25 ^Cab^	96.48 ± 11.82 ^Ca^
	C	0.82 ± 0.08 ^Ba^	0.72 ± 0.18 ^ABb^	0.22 ± 0.06 ^Aa^	0.09 ± 0.00 ^ABa^	236.35 ± 13.69 ^Cb^	67.99 ± 6.72 ^Ba^
	D	0.77 ± 0.14 ^ABa^	0.87 ± 0.04 ^Bb^	0.23 ± 0.01 ^Aa^	0.04 ± 0.00 ^Aa^	208.49 ± 25.52 ^BCab^	26.32 ± 4.88 ^Aa^
1-decanol	A1	0.89 ± 0.12 ^Ab^	0.57 ± 0.16 ^Aa^	0.17 ± 0.05 ^Aa^	0.09 ± 0.00 ^Aa^	303.77 ± 23.14 ^Bbc^	85.39 ± 11.52 ^Aa^
	A2	2.27 ± 0.17 ^BCc^	0.30 ± 0.05 ^Aa^	0.29 ± 0.04 ^Ba^	0.11 ± 0.01 ^Aa^	342.07 ± 25.15 ^Cc^	103.52 ± 14.01 ^Aab^
	B	1.41 ± 0.34 ^Ac^	1.06 ± 0.38 ^Cb^	0.13 ± 0.05 ^Aa^	0.16 ± 0.014 ^Aa^	228.05 ± 34.49 ^Aa^	165.68 ± 40.05 ^Bab^
	C	1.95 ± 0.46 ^Bn^	0.55 ± 0.34 ^Aa^	0.21 ± 0.15 ^ABa^	0.21 ± 0.2 ^Ba^	284.78 ± 36.68 ^Bbc^	182.51 ± 42.90 ^Bab^
	D	2.44 ± 0.28 ^Cb^	0.31 ± 0.02 ^Aa^	0.30 ± 0.02 ^Bab^	0.33 ± 0.01 ^Ca^	330.00 ± 20.71 ^Cc^	300.40 ± 34.36 ^Cb^
2-phenylethanol	A1	0.38 ± 0.04 ^Ba^	0.29 ± 0.09 ^Aa^	0.28 ± 0.08 ^Aab^	0.01 ± 0.00 ^Aa^	349.39 ± 29.52 ^Bc^	12.93 ± 2.04 ^Aa^
	A2	0.36 ± 0.03 ^ABa^	0.28 ± 0.01 ^Aa^	0.30 ± 0.01 ^Aa^	0.02 ± 0.00 ^Aa^	345.83 ± 22.57 ^Bc^	15.00 ± 1.61 ^Ba^
	B	0.28 ± 0.03 ^Aa^	0.39 ± 0.08 ^ABa^	0.21 ± 0.08 ^Aa^	0.02 ± 0.00 ^Aa^	249.58 ± 13.76 ^Aab^	9.91 ± 0.94 ^Aa^
	C	0.27 ± 0.03 ^Aa^	0.58 ± 0.13 ^Ba^	0.26 ± 0.06 ^Aa^	0.01 ± 0.00 ^Aa^	252.04 ± 9.52 ^Ab^	10.85 ± 0.75 ^Aa^
	D	0.26 ± 0.01 ^Aa^	0.53 ± 0.09 ^Ba^	0.29 ± 0.05 ^Aab^	0.03 ± 0.00 ^Ba^	252.45 ± 5.25 ^Ab^	21.23 ± 0.82 ^Ca^

Note: Different capital letters in the same column indicate significant differences (*p* < 0.05) between different alcoholic beverages in the same category of higher alcohols; different lowercase letters in the same column indicate significant differences (*p* < 0.05) between different categories of higher alcohols in the same alcoholic beverage.

## Data Availability

The original contributions presented in the study are included in the article/Supplementary Material, further inquiries can be directed to the corresponding author.

## Supplementary Materials

The following supporting information can be downloaded at: https://www.mdpi.com/article/10.3390/foods13203316/s1, Figure S1. Pharmacokinetic parameters of individual higher alcohols after intragastric administration of different alcoholic beverages; Table S1. Detailed information of alcoholic beverages in this study. Table S2. Quantitative parameters of ethanol and higher alcohols in SIM mode.
