# Supplementary material for: Composition of Higher Alcohols in Different Alcoholic Beverages and Their Metabolic Dynamics in Bama Pigs
Source: Foods. 2024 Oct 18;13(20):3316. doi: 10.3390/foods13203316 (PMC11507985; doi:10.3390/foods13203316)
Supplement: Supplementary file 1 [file foods-13-03316-s001.zip › foods-3217222-supplementary.pdf]

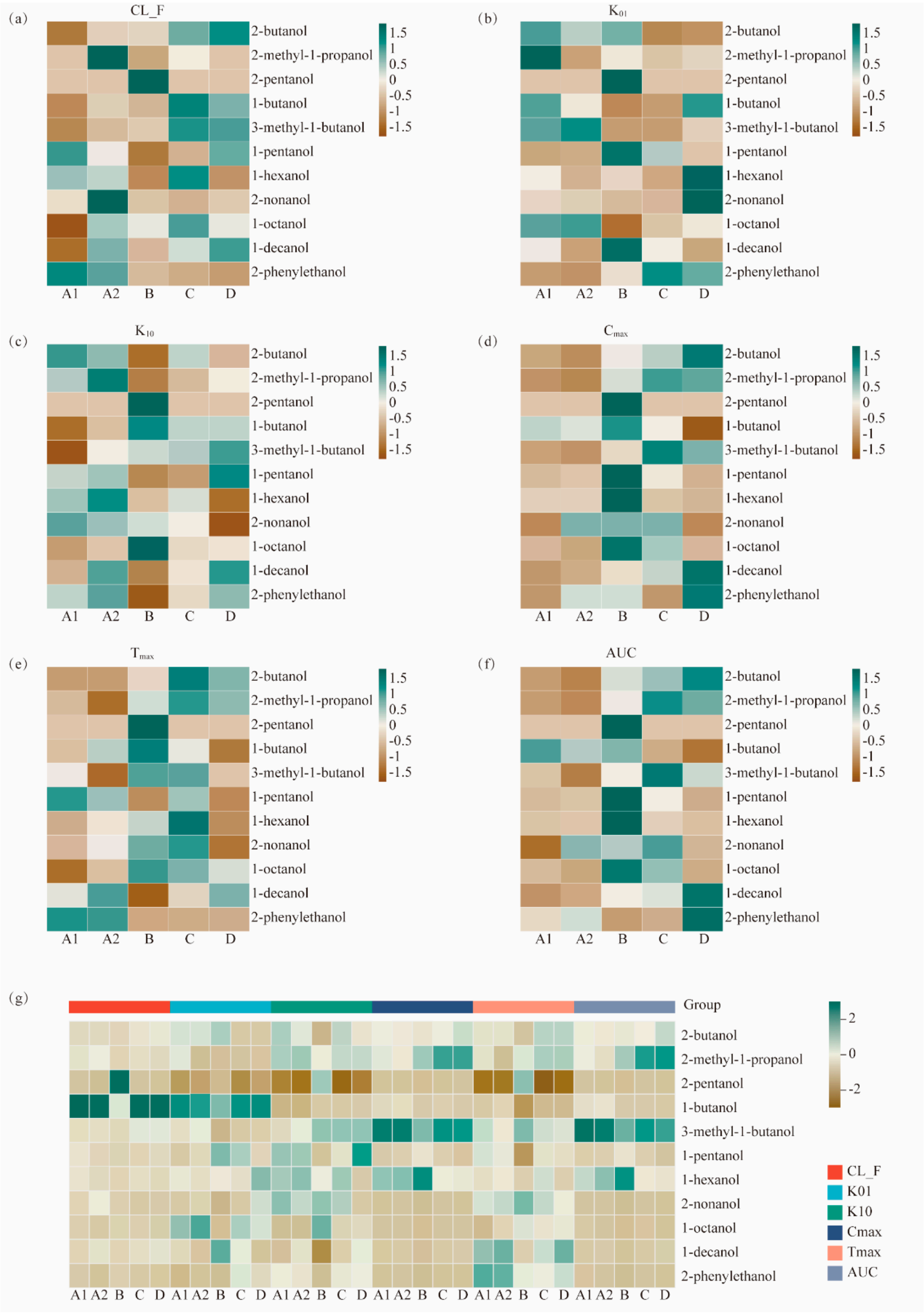

Figure S1. Pharmacokinetic parameters of individual higher alcohols after intragastric administration of different alcoholic beverages. (a)~(f) were the results of normalization according to rows and (g) were the results of normalization according to columns, respectively.

Table S1. Detailed information of alcoholic beverages in this study.

Samples	Flavor types	Raw materials	Region	Alcohol content (% vol)	Fermentation system	Distillation type	Aging type
Baijiu A1	Nong	Sorghum, maize, wheat	Luzhou, Sichuan	50.8%	Solid state	Pot still	Pottery jars
Lujiu A2	Nong	Tea extract, sorghum, maize, wheat	Luzhou, Sichuan	50.8%	Solid state	Percolation extraction	Pottery jars
Baijiu B	Nong	Sorghum, wheat, waxy rice, maize, corn	Deyang, Sichuan	52.0%	Solid state	Pot still	Pottery jars
Baijiu C	Jiang	Sorghum, maize	Luzhou, Sichuan	53.0%	Solid state	Pot still	Pottery jars
Baijiu D	Jiang	Sorghum, maize	Zunyi, Guizhou	52.0%	Solid state	Pot still	Pottery jars

Table S2. Quantitative parameters of ethanol and higher alcohols in SIM mode

No.	Retention time (min)	Compounds	CAS	Quantitative ion (*m/z*)	Regression equation	Linear range (mg/L)	R^2
0	3.56	ethanol	64-17-5	31*/45/46	$Y=4.596*10^{-6}X$	10-5000	0.9937
1	3.82	2-butanol	78-92-2	45*/59/85	$Y=1.477*10^{-5}X$	0.8-400	0.8259
2	4.59	2-methyl-1-propanol	78-83-1	41/43*/85	$Y=1.179*10^{-5}X$	0.8-400	0.9947
3	4.97	2-pentanol	6032-29-7	45*/55/73/43	$Y=2.944*10^{-5}X$	0.8-400	0.9981
4	5.45	1-butanol	71-36-3	41/56*/43	$Y=1.354*10^{-5}X$	0.8-400	0.9982
5	6.44	3-methyl-1-butanol	123-51-3	41/55*/57/70/39/42/43	$Y=1.799*10^{-5}X$	0.8-400	0.9988
6	7.15	1-pentanol	71-41-0	41/42*/55/57/70	$Y=1.867*10^{-5}X$	0.8-400	0.9976
7	8.93	1-hexanol	111-27-3	56*/69/41/43	$Y=3.305*10^{-5}X$	0.8-400	0.9983
8	9.58	3-octanol	20296-29-1	59*/83/101/55	$Y=3.922*10^{-5}X$	0.8-400	0.9944
9	10.84	1-heptanol	111-70-6	56/70*/41/43	$Y=3.365*10^{-5}X$	0.8-400	0.9992
10	12.07	2-nonanol	628-99-9	45*/69/55/98/111	$Y=5.600*10^{-5}X$	0.8-400	0.9966
11	12.83	1-octanol	111-87-5	56*/41/69/84	$Y=2.100*10^{-5}X$	0.8-400	0.9963
12	14.97	2-furan methanol	98-00-0	98*/81/53	$Y=2.808*10^{-5}X$	0.8-400	0.9995
13	16.96	1-decanol	112-30-1	70/55*/41/83/97/112	$Y=1.470*10^{-5}X$	0.8-400	0.9902
14	19.64	2-phenylethanol	60-12-8	65/91*/122	$Y=8.830*10^{-5}X$	0.8-400	0.9891

Note: * is the quantitative ion, and the others are auxiliary qualitative ions.
